# Place-Based FACT: Treatment Outcomes and Patients’ Experience with Integrated Neighborhood-Based Care

**DOI:** 10.1007/s10597-024-01277-4

**Published:** 2024-05-10

**Authors:** Welmoed van Ens, Sarita Sanches, Leonieke Beverloo, Wilma E. Swildens

**Affiliations:** 1 Altrecht Institute for Mental Health Care, Utrecht, The Netherlands; 2 Department of Nursing, Inholland University of Applied Sciences, Amsterdam, The Netherlands; 3 Avans University of Applied Sciences, Breda, The Netherlands

**Keywords:** Severe Mental Illness, Community Mental Healthcare, Integrated Care, Place-based care, Recovery, Routine Outcome Monitoring

## Abstract

Locating specialized mental healthcare services in the neighborhood of people with severe mental illnesses (SMI) has been suggested as a way of improving treatment outcomes by increasing patient engagement and integration with the local care landscape. The current mixed methods study aimed to examine patient experience and treatment outcomes in three Flexible Assertive Community Treatment (FACT) teams that relocated to the neighborhood they served, compared to seven teams that continued to provide FACT as usual from a central office. Routine Outcome Measurement (ROM) and care use data were analyzed to compare change in treatment outcomes for patients in place-based FACT (n = 255) and FACT as usual (n = 833). Additionally, retrospective in-depth interviews were conducted with twenty patients about their experience with place-based FACT. Quantitative analysis showed mental health admission days decreased more in place-based than FACT as usual, although this difference was small. Both groups showed improved quality of life, psychosocial functioning, and symptomatic remission rates, and decreased unmet and overall needs for care. There was no change over time in met needs for care, employment, and daily activities. Qualitative analysis showed that patients experienced place-based FACT as more accessible, a better safety net, a more personal approach, better integrated with other forms of care, involving their social network, and embedded in their neighborhood and daily environment. This study showed that location and integration matter to patients, and the long term impact of place-based FACT on treatment outcomes should be explored.

## Introduction

Despite substantial progresses since deinstitutionalization, people with severe mental illnesses (SMI) receiving care in the community continue to fare worse than the general population in many areas of life. Those once thought of as chronically mentally ill have achieved unexpected levels of personal, clinical and functional recovery outside of long-stay mental hospitals (McInerney et al., 2018; Priebe et al., 2002). However, in spite of this, people with SMI continue to report a lower quality of life (van Nieuwenhuizen et al., 2017), and high rates of loneliness and social isolation (Badcock et al., 2020; Fortuna et al., 2019; Kroon et al., 2021), unemployment (Hakulinen et al., 2019, 2020; Kortrijk et al., 2019), homelessness (Baptista & Eric, 2019; Craig & Timms, 2009), physical illness, (Nielsen et al., 2021a, 2021b) and a gap in life expectancy of roughly 10–20 years compared to people without SMI (Jayatilleke et al., 2017; Laursen et al., 2019; Lawrence et al., 2013; Walker et al., 2015).

A likely contributing factor to the persisting social and health inequalities faced by people with SMI living in the community is a lack of effective integrated care that addresses their interrelated needs in multiple domains (Craig & Timms, 2009; Lamb & Weinberger, 2020). The multifaceted nature of SMI necessitates professional support in several domains, ranging from physical and mental health to finances, employment and housing (Westen et al., 2020). While the mental hospital often functioned as a one-stop-shop for all the care deemed necessary, it must now be obtained from a multitude of organizations and institutions. Absence of care aimed at certain domains or lack of coordination between them may partially explain phenomena such as the under-treatment of physical illness in people with SMI (Swildens et al., 2016) and the over-involvement of the criminal justice system in managing acute psychiatric symptoms (Koekkoek, 2017; Lamb & Weinberger, 2020; Langton et al., 2021; Livingston, 2016). In addition to addressing stigma and prejudice (Brouwers, 2020; Evans-Lacko et al., 2013; Ilic et al., 2013), extensive and improved collaboration is needed to improve outcomes of people with SMI (Westen et al., 2020).

Although integrated models of care delivery have been shown to improve outcomes, it remains a challenge to coordinate the care and facilitate recovery for people with SMI. Evidence based care delivery models such as Assertive Community Treatment (ACT) incorporate multidisciplinary teams and a case management model as key components (Stein & Santos, 1998; Stein & Test, 1980). Flexible ACT (FACT) combines case management for stable patients with intensive ACT services when needed (van Veldhuizen, 2007). Compared to other care delivery models, FACT has been found to reduce the number of admissions or inpatient days, improve quality of life and reduce unmet needs (Drukker et al., 2008; Nielsen et al., 2021a, 2021b; Nugter et al., 2016; van Veldhuizen, 2007). FACT has been widely disseminated in the Netherlands, serving an estimated 70.000 patients (van Vugt et al., 2018) and is evolving to better engage the network of the patient in treatment (Tjaden et al., 2021). However, after several years of care, less than half of patients receiving ACT or FACT reach functional or clinical recovery (Huxley et al., 2021; Kortrijk et al., 2012; Nugter et al., 2016; Salzer et al., 2018). Although service delivery models such as FACT strive to provide integrated care, coordination remains a challenge in a complex fragmented care landscape (Trane et al., 2021).

Place-based care, an approach to service organization which situates mental health teams in close vicinity to patients’ homes, has been proposed to integrate services that operate in the same catchment area and improve patient engagement. The National Health Service (NHS) defined place-based care as “a multidisciplinary service across health and social care aligned with primary care networks” (NHS long term plan, 2019, p. 69) and listed integration as one of its primary aims. There is some evidence to support the assumption that proximity facilitates collaboration and engagement. For example, referrals from general practitioners (GP’s) to mental health and addiction services are most likely to succeed when services are co-located in the same practice (Bartels et al., 2004). These developments are particularly relevant to the Netherlands, one of the most densely populated countries in Europe, which is characterized by a uniquely high number of services operating in close proximity (van Veldhuizen, 2007). Paradoxically, this abundance makes for a fragmented care landscape, in which professionals are struggling to coordinate care across different life domains, and people with problems feel unsure where to turn for help. The need for initiatives that promote coordination is therefore evident, and the high density of services and service users lends itself well to collaboration within a small catchment area. At present, the FACT model fidelity criteria do not require FACT teams to be (co-)located in their catchment area alongside other health and social services (Westen et al., 2023). The FACT manual recognizes that co-location in the center of a neighborhood, preferably in the same building as other services (supported housing, social work, general practitioner) is ideal, but acknowledges that in reality many FACT teams are housed at some distance from their catchment area for practical, historical and financial reasons (van Veldhuizen & Bähler, 2013).

In addition to facilitating collaboration, place-based care is designed to provide location-based opportunities for recovery and reintegration in people’s own neighborhood. Place-based care is not merely delivered in people’s neighborhood, but embedded in the context of people’s daily lives, and regards their immediate environment as a source of opportunities and resources for recovery and reintegration (The Community Mental Health Framework for Adults and Older Adults, 2019). An environment that facilitates recovery has also been described by Rapp and Goscha as an ‘enabling niche’. Clinicians should strive to create enabling niches which facilitate and incentivize participation in work, recreation, and socializing, and allow people to be more than “just” patients (Rapp & Goscha, 2011). People with SMI report that ordinary spaces in their neighborhood, such as hair salons and parks, can serve as ‘enabling places’ that greatly facilitate their recovery (Duff, 2012). Moreover, ACT staff believe that facilitating positive experiences through activities and outings in the community can be an important catalyst for reintegration (Linz & Sturm, 2016). Since deinstitutionalization, emphasis has shifted away from relationships and activities contained within a psychiatric enclave, and people with SMI are encouraged to “build up safe havens all over the place” (Pinfold, 2000). Taken together, these findings show the immediate physical and social environment are crucial to recovery. A care team with a physical presence in the neighborhood could help patients connect to the resources for recovery available in their everyday environment and reintegrate into their communities in accordance with the post-deinstitutionalization ideal.

Based on these considerations, three FACT teams in the Netherlands implemented place-based care principles in 2016 and set up their offices in accordance with the recommendations provided by the FACT manual (van Veldhuizen & Bähler, 2013). Network partners including the municipality, sheltered and supported living services, recovery colleges run by people with lived experience, and family associations were consulted in the process. Firstly, FACT teams were rehoused so that care could be provided from offices in the immediate environment of patients. Teams that had previously operated from shared central offices at a distance of up to 5 miles from the catchment area they served were now situated within a few minutes walking or cycling distance from patients’ homes. Secondly, integration with other services was intensified in several ways. Catchment areas were redesigned to correspond to the boundaries of the catchment area of the social service teams. Caseworkers from the local supported living services in each neighborhood were integrated directly into the core interdisciplinary place-based FACT team, meaning they participated in regular team meetings. These changes resulted in a care delivery model that retained all the key elements of FACT with an added emphasis on place-based care.

The current study had two aims. The first aim was to assess whether the implementation of place-based FACT was associated with improved treatment outcomes in terms of number of met, unmet and overall needs for care, quality of life, symptomatic remission, psychosocial functioning, number of people with paid work and structured daily activities, and inpatient admission days. We expected that, compared to patients in FACT as usual, patients in place-based FACT would show a steeper rate of improvement on these outcomes. Since place-based FACT involves enhanced collaboration with the supported living team, we also expected the difference in rates of improvement between people receiving place-based FACT and FACT as usual to be enhanced if they received supported living services.

The second aim was to qualitatively explore the impact of place-based FACT on patients’ subjective experience of care, specifically focusing on the role of the high level of integration of previously fragmented services as well as the ultra-proximity of services, and identify themes that differentiate the experience of patients with place-based FACT from FACT as usual.

## Methods

### Design and Study Setting

The current study employed a longitudinal mixed method quasi-experimental design. It was conducted at a regional provider of specialized mental healthcare in the area of Utrecht in the Netherlands. It concerns ten existing FACT teams serving catchment areas of approximately 35.000 inhabitants each. These teams consisted of one or two psychiatrists, psychologists, experts by experience, and six to twelve casemanagers (mental health nurses and social workers). In addition to the core team, addiction experts and vocational rehabilitation coaches at times participated in team meetings. Three of the existing FACT teams implemented place-based FACT in September of 2016 while the remaining seven continued to deliver care as usual (FACT as usual). The teams that would participate in the pilot were selected at the management level by a steering group containing representatives of the mental health institute, the municipality, patients and their families. The teams were chosen because their catchment areas were challenging due to a high density of people living with severe mental illness and a relatively low socioeconomic status. Four of the FACT as usual and two of the place-based FACT teams served a catchment area in a predominantly urban area of Utrecht, while three of the FACT as usual and one of the place based team served the surrounding towns and villages.

### Sampling and Procedure

Place-based FACT was implemented in the pilot teams in September 2016. Data from ongoing ROM conducted between January 2015 and April 2018 were extracted for this study, spanning a baseline period of 1,5 years before and a follow-up period of 1,5 years after implementation. ROM questionnaires were completed by casemanagers at yearly intervals. At each measurement occasion patients were invited to participate in a concurrent voluntary assessment. Data from patients with at least one completed clinician assessments before and one after implementation were selected. Rates of change during the follow-up period (from the implementation in September 2016 until the end of the study period in April 2018) were compared between place-based FACT and FACT as usual while controlling for pre-implementation baseline scores recorded before September 2016. In addition to ROM data, care use data for the years 2015–2018 were extracted from electronic administrative care records.

Qualitative data were obtained retrospectively through a convenience sample of twenty in-depth semi-structured interviews conducted in 2018 and 2019 with patients from the pilot teams who could compare their experience from before and after the implementation of place-based FACT. We included patients who were able to give informed consent and converse in Dutch, and who had been receiving care as usual for some time before the transition to place-based FACT. Recruitment took place through flyers disseminated in waiting rooms and common areas and through staff of mental health teams, sheltered living teams and research assistants conducting routine outcome measurements. The interviews had a duration of approximately one hour (range 35—90 min) and took place at the patient’s home or at a community mental health center. We deemed saturation achieved when no new themes occurred for three consecutive interviews (Saunders et al., 2018).

### Instruments

**The Camberwell Assessment of Needs Short Appraisal Schedule** (CANSAS) is a semi-structured interview designed to measure (changes in) the number of met and unmet care-needs in 22 health and social-life domains during the past month (Phelan et al., 1995). Items are scored as no need (0), met need (1) or unmet need for care (2). The life domains assessed are accommodation, food, looking after the home, personal-hygiene, daytime activities, physical health, psychotic symptoms, information about treatment and diagnosis, psychological distress, safety of self, safety of others, alcohol, drugs, company, intimate relationships, sexual expression, childcare, basic education, telephone, transport, money, and benefits. The Dutch version contains an addendum with three additional items, namely: paid work, side effects of medication, and meaningful life and recovery (Drukker et al., 2010). The clinician-rated version was completed by casemanagers. Summary scores with a range of 0–25 were calculated for the number of overall needs, met needs and unmet needs for care. The interrater agreement between patients and staff is excellent for summary scores and test–retest reliability is acceptable (Phelan et al., 1995). The internal consistency of the CANSAS has been reported as poor (McCrone et al., 2000), but internal consistency was acceptable to good in the current study with a Cronbach’s alpha of 0.81 at baseline for unmet needs, 0.69 for met needs and 0.82 for total needs.

**Manchester Quality of life Short Assessment** (MANSA) is a self-report questionnaire for people with SMI that measures quality of life (Priebe et al., 1999). It contains twelve items on satisfaction in the domains of housing, housemates, daily activities, physical health, mental health, personal safety, social relationships, family relationships, partner relationship, sex-life, financial situation, and life overall. Answers are given on a Likert scale ranging from very dissatisfied (1) to very satisfied (7). Additionally, the MANSA contains four dichotomous items on the presence or absence of close friendship, social contact, victimization and perpetration. The summary score was computed as the sum of all 12 Likert scale item scores with a range of 0 to 84. The MANSA had good internal consistency with a Cronbach’s alpha of 0.83 at baseline in the current study.

**Health of the Nation Outcome Scale** (HoNOS) is a clinician-rated instrument that measures (clinical) symptoms and problems in psychosocial functioning over the previous two weeks (Mulder et al., 2004; Wing et al., 1998). It contains four subscales on behavioral problems, impairment, psychiatric symptoms and social problems and consists of 12 items. Items are rated as 0 (no limitations), 1 (minor problems, requiring no formal action), 2 (mild problems, requiring clinical intervention), 3 (moderate problems), 4 (severe to very severe problems). The total HoNOS score can range from 0–48. The Dutch version has been successfully tested for use among people with SMI (Mulder et al., 2004) and had a Cronbach’s alpha of 0.76 at baseline in the current study.

**Symptomatic remission** was calculated from the HoNOS items psychotic symptoms, mood, and other psychiatric symptoms. Participants who scored no higher than 0 or 1 on all three items were classified as in remission.

**Sociodemographic Characteristics** were recorded on age, gender, level of education, independent living (yes/no), employment status, and whether or not participants engaged in structured daily activities.

**Care Use Data** were extracted from administrative care records for the period of January 2016 through March 2018, specifically the number of monthly inpatient mental health admission days, the number of contacts with a casemanager or psychiatrist, and whether a patient was still receiving specialized mental healthcare by the end of this period. Average number of monthly admission days was calculated for the baseline period (January 2016 through September 2016) and the follow-up period (October 2016 through March 2018). Additionally, average number of monthly contacts with the service was calculated for the follow-up period.

**Topic lists** were used to guide the semi-structured qualitative interviews (available on request). The topic list was based on the primary goals of place-based FACT as formulated by FACT teams, patient and family organizations, and care network partners working in the neighborhood as well as literature on personal, functional and clinical recovery. Interview topics included recovery, participation, support from others, accessibility of care, care across multiple domains, and collaboration and integration of care. If participants had received professional support in these areas, they were asked if and how the support had changed since the introduction of place-based FACT. Additionally, they were asked about advantages and disadvantages, their overall satisfaction, and points of improvement regarding the support provided by place-based FACT in these areas.

### Analysis

Quantitative data were analyzed using SPSS 28 and HLM6. Two-level hierarchical regression models were used to analyze the difference in improvement in treatment outcomes between the intervention and control group. Measurements (level 1) were nested in persons (level 2). There were not enough treatment teams to introduce a third level in the model (Hox et al., 2017). Therefore, differences between teams were controlled for using dummies representing the place-based FACT teams and a dummy to indicate whether the FACT as usual teams were serving an urban versus comparatively rural catchment area. In order to compare the difference in recovery rates between FACT as usual and place-based FACT from the moment of implementation onwards, time was split into time during the baseline and time follow-up period, both expressed in years. Time during baseline covered the period before implementation when patients in both groups were receiving FACT as usual. During follow-up, the pilot teams delivered place-based FACT while the patients in the comparison group continued to receive FACT as usual. The difference in change was then analyzed through the interaction between treatment group and time during the follow-up period. Predictors were added to the model sequentially. The final model for each outcome is reported in this paper.

Admission days were compared for the entire baseline and follow up period to make use of all available data extracted from administrative records, which spanned the period of January 2016 through March 2018. Number of average monthly admission days was compared using a repeated measures ANOVA with an interaction between time period (baseline or follow-up) and intervention group.

Finally, a Chi-square analysis was conducted to determine whether the number of people who had left specialized mental healthcare, either due to discontinuation or discharge, by the end of the study period differed between place-based FACT and FACT as usual.

Due to the naturalistic clinical setting of the study, availability of data varied between patients. Clinician-rated instruments were completed more frequently than the patient-rated MANSA. In accordance with the standard procedures for the instruments used, simple person-mean imputation was applied when less than 20% of items in an instrument were missing, and summary scores were treated as missing if more than 20% of items were missing. Listwise deletion was applied separately for each analysis in HLM and sample sizes for each outcome varied accordingly depending on the number of valid outcome measurements. Alpha was set at 0.05 for all statistical tests.

Qualitative interview data were transcribed and analyzed by four researchers in Nvivo (released 2020). A thematic analysis was conducted (Braun & Clarke, 2006). Interviews were first coded independently by two researchers and then discussed until consensus about naming was reached. Regular discussion between the researchers and member checks were performed to improve quality of data analysis. Attention to negative cases was used to enhance validity.

### Ethical Considerations

Informed consent was obtained from patients who participated in the semi-structured interviews. Quantitative data were extracted from existing clinical records, which did not require informed consent under Dutch law during the study period. However, patients could choose to opt out of having their data used for research purposes, in which case their data were excluded. This study was approved by the institutional ethical medical review board of Altrecht (CWO-1617).

## Results

### Sample Description

The final sample for the quantitative analysis consisted of 1088 patients, 833 of whom continued to receive FACT as usual from the seven control teams while 255 received place-based FACT from three pilot teams. Baseline sample descriptives are provided in Table 1, which shows the majority of the participants had a diagnosis of schizophrenia or psychotic spectrum disorder. The average HoNOS score was 8.45 (*SD* 5.86, range 0–34.91), indicating a moderately severe impairment in psychosocial functioning, and 32% scored above 11, which is the severity threshold at which intensified care or inpatient care could be considered (Nugter et al., 2012). There were significantly more people in sheltered or supported living in the place-based FACT than the FACT as usual group, but the groups did not differ on any other baseline characteristics. The average number of monthly contacts between patients and clinicians did not differ significantly between FACT as usual (*M* = *2.91, SD* = *3.47*) and place-based FACT (*M* = *2.78, SD* = *2.72*, t(1080) = 0.530, p = 0.597) during the intervention phase of the study. There was no significant difference in the number of people who had left care by the end of the study period in the place-based group and the FACT as usual group (79.6% and 76.4% respectively, χ^2^ (1) = 0.298, p = 0.585).

**Table 1:** Sample Descriptives at Baseline

Patient characteristics	FACT as usual	Place-based FACT	Total sample
Age (M. SD)	45.47 (10.43)	44.59 (10.14)	45.28 (10.36)
HoNOS (M. SD)	8.42 (5.93)	8.56 (5.67)	8.45 (5.86)
% Female	34.8	30.2	33.7
% Psychotic disorder^a^	79.3	82.4	80.0
% Non-western migration background^b^	37.1	39.8	37.7
% Long-term relationship	26.3	23.5	25.6
% Sheltered or supported living^c^	20.8	31.1	23.2
% In paid employment	13.0	10.6	12.4
% Low level of education^d^	53.2	48.9	52.2
% One or more mental health admissions % One or more mental health admissions	24.0	25.5	24.4

The characteristics reported here are those of the overall sample (N = 1088)

^a^Patients with a primary diagnosis of schizophrenia or other psychotic spectrum disorder

^b^Patient or one or both parents born in non-western country

^c^There was a significant difference in the number of people living in sheltered living facilities or receiving supported living services between the control and intervention group (χ2 (1) = 15.10. p < .001)

^d^No qualifications beyond lower level secondary education

The average number of routine outcome measurements conducted during the study period was 3.10 (*SD* = *0.60, range* = *1–6*) per patient with an average of 11.52 (*SD* = *2.32*) months between each measurement. The average follow-up time after the implementation of place-based FACT was 10.28 months (*SD* 4.08) with a maximum of 17.88 months. Of the 3167 assessments included in the study, 880 (26.1%) included self-report measures from the voluntary patient assessment. 45.3% of patients included in the study participated in at least one assessment. Compared to patients who never participated, those who participated in a voluntary assessment interview had a lower HoNOS score (7.36 (*SD* 5.18) versus 9.39 (*SD* 6.24) respectively, t(1053) = 5.678, p < 0.001) and were less likely to have a non-western migration background (28.2% versus 45.5%, χ ^2^ (1) = 35.67, p < 0.001).

### Quantitative Results

The results of the multilevel analyses for each outcome measure are found in Table 2 and 3. There were no significant interactions between intervention and time during follow-up, meaning there was no difference in the rate of improvement between patients receiving FACT as usual and patients receiving place-based FACT on any of the outcomes. Irrespective of treatment group, number of unmet and overall number of care needs (CANSAS) decreased significantly during the baseline and follow-up period. Quality of life (MANSA) increased significantly across groups during the baseline period but not during follow-up. The number of people in symptomatic remission increased significantly during follow-up but not during the baseline period, and problems in psychosocial functioning (HoNOS) decreased significantly during follow up but not during baseline. Number of met needs, paid employment and daily activities did not change significantly over time during the baseline nor follow-up period.

**Table 2:** Results of Logistic Multilevel Regression Analyses

	Paid work		Structured daily activities		Symptomatic Remission	
	N = 1076		N = 1076		N = 1076	
	OR	95% CI	OR	95% CI	OR	95% CI
Intercept	0.228*	[0.197, 0.263]	0.663*	[0.556, 0.792]	0.661*	[0.557, 0.784]
Time baseline	1.019	[0.951, 1.091]	1.080	[0.976, 1.195]	1.035	[0.918, 1.167]
Time follow-up	1.088	[0.973, 1.216]	1.154	[0.983, 1.354]	1.239*	[1.039, 1.476]
Place-based FACT team 2 (urban)	0.964	[0.758, 1.228]	0.780	[0.579, 1.050]	0.776	[0.577, 1.044]
Place-based FACT team 3 (rural)	1.049	[0.842, 1.306]	1.050	[0.798, 1.383]	0.726*	[0.558, 0.945]
FACT as usual (rural)	1.070	[0.947, 1.210]	1.073	[0.930, 1.238]	0.982	[0.864, 1.116]
Treatment group	0.820	[0.642, 1.048]	0.831	[0.609, 1.132]	1.309	[0.973, 1.761]
Supported living	0.675*	[0.544, 0.838]	1.257	[0.932, 1.695]	0.918	[0.692, 1.217]
Treatment*supported living	1.021	[0.676, 1.543]	0.899	[0.509, 1.587]	0.721	[0.417, 1.246]
Time*supported living	0.860	[0.694, 1.066]	1.108	[0.797, 1.540]	0.768	[0.542, 1.088]
Time*treatment	1.132	[0.875, 1.464]	0.969	[0.683, 1.374]	0.844	[0.579, 1.232]
Time*treatment*supported living	0.915	[0.626, 1.337]	1.164	[0.619, 2.189]	1.211	[0.620, 2.364]

^*^significant at the alpha = .05 level

**Table 3:** Results of Linear Multilevel Regression Analyses

	CANSAS total		CANSAS unmet		CANSAS met		HoNOS		MANSA	
	N = 1048		N = 1048		N = 1048		N = 1076		N = 487	
	B	SE	B	SE	B	SE	B	SE	B	SE
Intercept	7.901*	0.188	2.691*	0.133	5.167*	0.145	7.895*	0.257	60.976*	0.818
Time baseline	-0.293*	0.112	-0.284*	0.087	0.005	0.101	-0.163	0.146	1.816*	0.520
Time follow-up	-0.321*	0.160	-0.246*	0.112	-0.100	0.145	-0.542*	0.204	0.453	0.693
Place-based FACT team 2 (urban)	0.907*	0.368	0.111	0.251	0.708*	0.302	0.449	0.454	2.179	1.364
Place-based FACT team 3 (rural)	-0.684*	0.321	-0.032	0.235	-0.652*	0.237	-0.027	0.394	-1.869	1.292
FACT as usual (rural)	-0.470*	0.151	-0.247*	0.105	-0.306*	0.112	-0.472*	0.201	0.337	0.622
Treatment group	0.904*	0.353	0.434	0.257	0.500	0.268	0.072	0.462	-0.513	1.453
Supported living	2.331*	0.335	-0.260	0.232	2.618*	0.272	0.976*	0.458	-1.455	1.399
Treatment*supported living	-0.696	0.630	-0.668	0.443	-0.062	0.524	0.065	0.820	3.912	2.479
Time*supported living	0.791*	0.356	0.774*	0.261	0.012	0.298	0.817	0.457	-2.009	1.530
Time*treatment	-0.055	0.314	-0.061	0.261	0.051	0.300	0.075	0.496	-1.240	1.356
Time*treatment*supported living	-0.705	0.664	-0.040	0.491	-0.617	0.635	0.176	0.882	-1.481	3.069

^*^significant at the alpha = .05 level

Compared to those living independently, patients receiving supported living services scored significantly higher on met and overall care needs and on the HoNOS and were less likely to have paid work or meet criteria for symptomatic remission. There was also a significant interaction effect of supported living and time during follow-up on the outcomes met and overall care needs, showing a smaller reduction in needs for people in supported living. There were no significant interaction effects between place-based FACT and supported living or between intervention group, supported living and time during follow-up, meaning there was no difference in the change in outcomes for people in supported accommodation who received place-based FACT and FACT as usual.

**Fig. 1:**
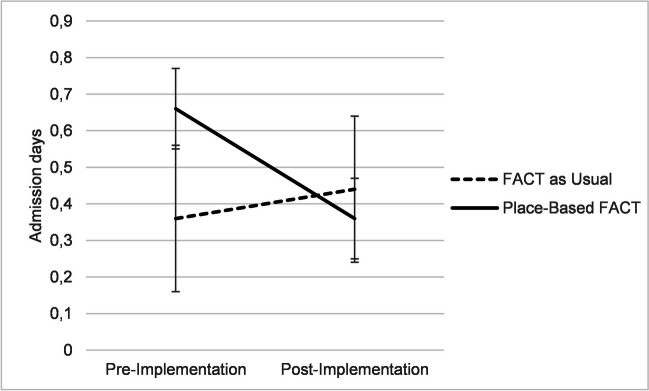
The interaction effect of time and intervention group on mean monthly admission days

The results of the repeated measures ANOVA of the effect of time and intervention group on average admission days per month in the period before and after the implementation of place-based FACT showed there was no significant overall change in admission days over time (F(1, 1086) = 2.64, p = 0.105) or overall significant difference between the two treatment groups (F(1, 1086) = 1.42, p = 0.233). There was however a very small but significant interaction effect of time by intervention (F(1) = 7.12, p = 0.008, η^2^ = 0.007) which is illustrated in Fig. 1. The average number of admission days per month decreased from 0.66 (95% CI [0.47-0.86]) pre-implementation to 0.36 (95% CI [0.16-0.56]) post-implementation in the place-based FACT group, while it increased slightly from 0.36 (95% CI [0.26-0.47]) to 0.44 (95% CI [0.32-0.55]) in the FACT as usual group.

### Qualitative Results

Sixteen men and four women participated in a semi-structured in-depth interview. They ranged in age from 32 to 66 years old with an average of 47.9. Ten of these patients had been diagnosed with schizophrenia or psychotic spectrum disorder, six with schizoaffective disorder, two with a mood disorder without psychotic symptoms, one with a personality disorder, and one had an unknown diagnosis. Patients had been receiving place-based FACT for approximately 1.5 to 2.5 years at the time of the interview. Ten of the participants also received supported living services.

Six themes pertaining to place-based FACT were identified, namely: accessible, safety net, personal, integrated, neighborhood-based, and network involvement.

#### Accessible

Participants were very pleased with the proximity of the new offices to their homes.

Well, I just hop on my bike and I’m there within five minutes. And in this way I can also take my responsibility.

This proximity enabled more frequent house calls as well as spontaneous visits to the office as needed. Sometimes participants would pop in for a chat, a cup of coffee or to use the internet. Help could be obtained or provided quickly when needed.

If I’m not doing well and I’m at the mall, then I can walk over and ring the doorbell and go, I’m not doing well. So in that respect the step has gotten a lot smaller.

[…] if I don’t manage to come here, they can be at my doorstep in no time.

The only downside in terms of accessibility was that the availability of the team was often limited to office hours and arrangements had to be made for the patient to be supported elsewhere if they needed help outside these times.

One participant felt that place-based FACT helped prevent people with mental health problems from causing a disturbance on the street, because they could now easily drop in to talk instead. This participant made explicit what the effect of accessibility can be on recovery, namely to prevent crisis and relapse, and mitigate their impact when they do occur.

#### Safety Net

Participants experienced an increased sense of safety and security due to the accessibility and proximity of the neighborhood-based team. They felt reassured by the idea that someone would be right there for them if they needed help.

It mainly comes down to a feeling of safety. Yeah. That there’s someone there.

The biggest thing is just the feeling of safety, that it’s close by.

During difficult times, the team acted as a safety net that patients could rely on. Being close by and familiar with the area enabled staff to attend situations where patients found themselves in acute distress, preventing the situation from escalating into a potentially traumatizing incident or public disturbance.

I was at the [supermarket], but I was feeling a little confused, all of a sudden, and then I called [casemanager], […] and he put an arm around me, and he said to everyone, he looked them in the eye and he said “yeah, he’s a little confused, leave him be, why don’t you get out of the way.” I was completely lost for a moment, and he took me [to the FACT office]. That is compassion.

#### Personal

Most patients felt that neighborhood-based care entailed a more personal welcoming approach. They appreciated seeing familiar faces when they came to the office and being recognized by the staff as a result of working with a smaller team. They felt staff knew and remembered their story. Because of their close acquaintance with patients, staff were able to spot warning signs and offer help pro-actively. This approach generally made patients feel seen, heard and supported.

[…] with the casemanager in [the old FACT team] I felt that there was a lot more distance between us. That I was one of many. And that feeling […] that they are committed and involved. I really appreciate that.

One participant actually preferred the relative anonymity of the central location over the personal approach of the neighborhood-based team.

#### Integrated

In addition to providing mental health support, staff helped patients address issues they encountered in other areas of life, including employment, daily activities, finances and administration, education, social relationships and their living situation. Practical hands-on support, ranging from doing groceries together to opening and sorting mail, was often provided directly by their casemanager from the place-based FACT team or supported living coach. While FACT teams have always aimed to provide integrated care addressing care needs in multiple domains, some participants noted that the integration of the place-based FACT team with the supported living team broadened the number of resources readily available to them.

What I do notice is that her toolbox is much larger. […]A few months ago we were talking about that I am having wild dreams, with certain recurring themes. And then [the supported living coach] said “If you like, I can contact the psychologist, because they are right next to us now.” There is no distance there anymore.

Additionally, the integration between the supported living and place-based FACT team improved communication between the different care providers involved in treatment. Information sharing and continuity of care were facilitated through co-locating services and joint meetings.

[…] because they’re all close together and consult together I do think it is an improvement and better than [the old FACT team].

#### Neighborhood-Based

The transition to neighborhood-based care was difficult for some participants, especially when it involved a change in casemanager. The change felt sudden and like a big step for them. One participant worried vulnerable people might relapse. Another downside was that their relationship with their care team was now tied to their place of residence, and moving to a different neighborhood could mean a change in care team. However, once settled, patients felt there were advantages to receiving care in their own neighborhood. They felt more at ease visiting the office in their own familiar surroundings.

But I find it, I don’t know how to describe it, but I think it’s better. And familiar. Also, because it is in your own neighborhood. Then you feel yourself at home.

More personal, yes. Maybe because it’s in your own environment, your home is here. You live your life here. And all of that is familiar.

The team could now provide support in their immediate environment, which was previously rendered impractical by the distance. It became possible for casemanagers to connect patients to services and resources in the community simply by accompanying them.

They also took me [to the pharmacy] a couple of times and showed me where I had to be, and then I could do it myself.

In this way, being able to do more things together with their coach or casemanager in their own environment allowed participants to develop skills and confidence, ultimately promoting their independence and personal recovery.

Additionally, they felt that there was less stigma attached to visiting a small office in their own neighborhood. Compared to the central offices, which had been co-located with inpatient wards in a large complex in the city center that was well-known for being a psychiatric institution, the place-based FACT team felt more “normal”.

#### Network Involvement

The place-based FACT team involved other formal and informal care-providers in the networks of the patients in their care. Participants felt staff were committed to serving their communities and working with their network rather than just the individual patient. Several mentioned collaboration had improved.

I think the threshold is lower, my mother will for example just call [casemanager], then the way it went at the FACT. […] here there is a whole floor. All the people walking around here serve just one neighborhood. That’s quite a difference.

Other examples included regularly gathering multiple family members together for a joint meeting with the patient to discuss their situation, or accompanying a patient on a GP visit to explain their mental health condition and advocate for their needs. In some cases, the casemanager communicated with other care providers and institutions on the patient’s behalf, for example by calling to set up a doctor’s appointment or applying for social housing.

Well, they applied to a fund for me, furnishing costs. […] my previous casemanager, she applied for a declaration of urgency.^1^ She has also often written good letters for me to institutions and the like.

Although the team took an active role in referring patients and streamlining communications, some patients felt that it was still unclear who they needed to approach for certain issues.

## Discussion

This study evaluated the impact of relocating FACT teams to operate in the immediate daily environment of the patient and redesigning catchment areas to promote integration with other local services. We hypothesized that patients would report a largely positive experience with place-based FACT and treatment outcomes would be more favorable than for FACT as usual. There was a small difference in the change in admission days in favor of place-based FACT, where admission days decreased relative to the FACT as usual group. Rates of improvement on other treatment outcomes did not differ between patients who received place-based and FACT as usual. However, patients in both groups did show a decrease in unmet and overall needs, an improvement in quality of life, and an increase in symptomatic remission, either during baseline, follow up or both. There was no change in the number of people with paid work and daily activities and number of met needs during the study period. Patients receiving supported living services were hypothesized to benefit most from place-based FACT. Though treatment outcomes for patients receiving supported living services were not superior in the place-based FACT group, patients did report improved collaboration between mental health and supported living services in the place-based FACT group in the qualitative interviews. A qualitative analysis of in-depth interviews showed that patients generally favored place-based FACT. They described it as being more accessible, providing a better safety net, entailing a more personal approach, being better integrated with other forms of care including supported living services, involving their social network, and being embedded in their neighborhood and daily environment.

The treatment outcomes demonstrate that patients improved similarly under the care of regular and place-based FACT-teams, with tentative evidence for a small reduction in admission days in place-based FACT. This would suggest that place-based FACT either prevents patients from deteriorating to the point of requiring hospitalization, or makes it possible to continue to care for them in their own homes during times of crisis which would have otherwise necessitated (longer) inpatient care. This may be the result of improved accessibility and engagement, corresponding to previous findings that patients who live further away from services are less likely to receive outpatient care and have less frequent sessions when they do (Schmitt et al., 2003; Zulian et al., 2011). Other studies conducted in these place-based FACT teams in the period following implementation also noted extensive collaboration with GP’s, social services, and community police officers in the neighborhood, as well as teams serving as a point of contact for concerned neighbors (Muusse et al., 2021a, 2021b). Collaboration between these parties may have prevented escalation. Although encouraging, the difference in admission days should be interpreted with caution. The quasi-experimental design prohibits causal inference, and, more importantly, the difference was very small and may not be clinically significant. Nevertheless, a 3.6 day reduction in average yearly admission days in the place-based FACT group would have amounted to a yearly cost savings of approximately €1100 per patient during the time of the study (Sanches et al., 2022).^2^ If substantiated by further research, the potential of place-based FACT to reduce admission days could contribute to reducing the substantial costs associated with hospitalization as well as the accompanying loss of autonomy and disruption to the person’s life.

The equivalent recovery rates of people in supported living receiving place-based and FACT as usual in the current study show that the high level of integration between services did not translate to a short-term clinical benefit. Previous research has found that patients who require ACT or sheltered living services also require hospitalization more often than those who use neither service, but that patients who receive a combination of both are hospitalized less often (Buchtemann et al., 2016). This enhanced combined effect was not found in the current study. Patients receiving supported living services in the current study were already receiving FACT services prior to the implementation of place-based FACT, which may have created a ceiling effect. Alternatively, a longer follow up period than 1.5 years may be required to make a difference in the clinical outcomes of people who require this level of care.

The predominantly positive themes that emerged in the qualitative analysis in relation to place-based FACT relate meaningfully to previous research on what matters in the treatment of (severe) mental illness. Patients are more satisfied with support from a care provider working in their community (Stamboglis & Jacobs, 2020). The proximity of the new offices enabled staff to provide prompt assistance in patients’ everyday environment, which people with psychosis rank among the most important aspects of care (Sterk et al., 2013). The theme safety net has been identified as an important reason for staying in treatment (Pettersen et al., 2014). Furthermore, the personal approach that patients experienced in place-based FACT resembles characteristics of strong therapeutic alliance, which in turn has been related to treatment adherence and outcome (Martin et al., 2000; McCabe & Priebe, 2004). Taken together, the findings from the qualitative analysis show that patients prefer place-based FACT not merely because it is more conveniently organized for them, but because they perceive a positive impact on their recovery. Our findings suggest that a place-based approach can cultivate the conditions needed for them to easily access help, take responsibility, and achieve a higher degree of independence and stability, preserving their dignity and autonomy. Through these pathways, place-based FACT may contribute to aspects of personal recovery such as empowerment, and perhaps in time to improved clinical outcomes beyond the follow-up period of this study.

### Strengths and Limitations and Suggestions for Future Research

The primary strengths of the current study were its naturalistic clinical setting, multi-informant mixed methods approach and the presence of a comparison group. The quasi-experimental design contributed to the ecological validity of the study. As no exclusion criteria applied and patients were not reassigned to treatment conditions through randomization, our study offers a realistic insight into the results of relocating existing FACT teams to be closer to their patients and network partners. The combination of clinician rated and patient reported outcomes and experiences with care use data provided a more complete picture. This is especially important as the subjective experience of patients with treatment has often been wrongly neglected in the past (Ruggeri et al., 2003). Future research should also explore how place-based FACT impacted professionals. Research on professionals’ experience with interprofessional collaboration in place-based FACT by the authors of the current study is ongoing.

Limitations of this naturalistic approach were the inability to make causal inferences because pilot teams were not selected randomly, its reliance on the availability of clinical data and potential bias due to missing data, and the limited follow-up period. Place-based FACT teams served neighborhoods with a high density of people living with severe mental illness and a relatively low socioeconomic status compared to most of the FACT as usual teams. Additionally, patients who voluntarily participated differed from those who did not on several baseline characteristics. Participants in the qualitative interviews made more use of supported living services. They may have been more firmly embedded in the care system and had a greater need for enhanced collaboration, leading to a more positive view of place-based FACT. On the other hand, patients with a migration background and more severe symptoms appeared to be underrepresented in the quantitative data. Persistent efforts are needed to improve the representation of underserved populations in mental health research. Finally, the follow-up time in the current study was 1,5 years. Though a considerable follow-up period, certain treatment goals, such as rebuilding a social network, may take more years to achieve, and a longer follow-up time may be needed to measure the full impact of place-based FACT on these outcomes.

### Conclusions

Taken together, the slight reduction in admission days and positive subjective experiences of patients within the first 1,5 years following implementation suggest that location and integration play a role in determining the effectiveness of FACT. Through working closely with the patient and their network in their own environment, place-based FACT teams may be able to support patients more satisfactorily and reduce costly and disruptive hospitalizations. More research with a longer follow-up time is needed to determine if the reduction in admission days is consistent and clinically relevant and whether the improved subjective experience of care will translate to improvements on other clinical outcomes in the long run. The fact that patients largely prefer to receive care from a dedicated team situated in their own neighborhood, combined with the absence of any evidence of heightened deterioration or drop-out following the transition, suggests that relocating teams that operate from a centralized location is both feasible and desirable. This warrants renewed attention for the implementation of the recommendation made in the existing FACT manual by van Veldhuizen and Bähler to (co-)locate offices in the center of their catchment area alongside other health and social services (van Veldhuizen & Bähler, 2013). A more explicit description of what constitutes a reasonably close location of offices relative to a catchment area, taking into account differences in population density and other regional factors, could be developed in future FACT guidelines, and incorporated in the model fidelity criteria.

## Footnotes

1 A declaration of urgency is issued to people in inadequate housing or living situations or who are at risk of becoming homeless, and gives them priority over other people on the long waiting list for social housing.

2 Calculated according to the price-point indexed for 2017, halfway through the follow-up period, which was €308,95 for each inpatient day in a psychiatric setting.
